# *DACT1*, an antagonist to Wnt/β-catenin signaling, suppresses tumor cell growth and is frequently silenced in breast cancer

**DOI:** 10.1186/bcr3399

**Published:** 2013-03-12

**Authors:** Xuedong Yin, Tingxiu Xiang, LiLi Li, Xianwei Su, Xingsheng Shu, Xinrong Luo, Jianbo Huang, Ying Yuan, Weiyan Peng, Michael Oberst, Kathleen Kelly, Guosheng Ren, Qian Tao

**Affiliations:** 1 Molecular Oncology and Epigenetics Laboratory, The First Affiliated Hospital of Chongqing Medical University, 1 Youyi Road, Chongqing 400016, China; 2 Cancer Epigenetics Laboratory, Department of Clinical Oncology, Sir YK Pao Center for Cancer and Li Ka Shing Institute of Health Sciences, The Chinese University of Hong Kong and CUHK Shenzhen Research Institute, Shatin, NT, Hong Kong; 3 Signal Transduction Section, National Cancer Institute, National Institutes of Health, 9000 Rockville Pike, Bethesda, MD 20892, USA; 4 Cancer Center, Dalian Medical University, 9 Lvshun Road South, Dalian 116044, China

## Abstract

**Introduction:**

Aberrant activation of Wnt/β-catenin signaling plays an important role in the pathogenesis of breast cancer. *DACT1 *(Dapper/Frodo) has been identified as involved in antagonizing Wnt/β-catenin signaling through interacting with Dishevelled (Dvl), a central mediator of Wnt signaling, whereas its role in breast tumorigenesis remains unclear.

**Methods:**

We examined *DACT1 *expression in breast cancer cell lines and primary tumors with semiquantitative or quantitative RT-PCR and immunochemistry, and further evaluated the promoter methylation of *DACT1 *with methylation-specific PCR (MSP). We also explored the tumor-suppressive functions of DACT1 *in vivo *and *in vitro*, and its related mechanism in breast cancer.

**Results:**

We identified *DACT1 *as a methylated target in our breast cancer epigenome study. Here, we further investigated DACT1 expression in multiple breast cell lines and primary tumors, and further studied its function and molecular mechanisms. We found that *DACT1 *expression was silenced in eight (88.9%) of nine breast cancer cell lines, and its protein levels were obviously reduced in breast tumors compared with paired surgical-margin tissues. Promoter CpG methylation of *DACT1 *was detected in five (55.6%) of nine breast cancer cell lines and 40 (29.9%) of 134 primary tumors, but not in surgical-margin tissues and normal breast tissues. Demethylation treatment of breast cancer cell lines restored *DACT1 *expression along with promoter demethylation, suggesting that an epigenetic mechanism mediates *DACT1 *silencing in breast cancer. Functional assays showed that ectopic expression of DACT1 could inhibit breast tumor cell proliferation *in vivo *and *in vitro *through inducing apoptosis, and further suppress tumor cell migration through antagonizing the Wnt/β-catenin signaling pathway.

**Conclusions:**

Our study demonstrates that DACT1 could function as a tumor suppressor but was frequently downregulated in breast cancer.

## Introduction

Breast cancer is the leading cause of cancer death among females [1], and results from accumulated genetic and epigenetic alterations of various cancer genes, including tumor-suppressor genes (TSGs) and oncogenes [2]. Epigenetic alterations, especially CpG promoter methylation, play important roles in the initiation and progression of multiple cancers including breast cancer. Hypermethylation of CpG island (CGI) in the promoter regions of TSGs is an alternative mechanism for TSG silencing and could occur early in tumorigenesis, thus serving as a promising tumor marker for breast cancer diagnosis [3]. Aberrant promoter methylation of some TSGs, such as *RASSF1A*, *BRCA1*, *TWIST*, *Cyclin D2*, and *p16*, has been shown to be good biomarkers for the early detection or for a therapeutic target in breast cancer [4,5].

Wnt/β-catenin signaling plays an important role in multiple tumorigenesis, including breast cancer [6]. Epigenetic silencing of negative regulators of WNT signaling is crucial for the aberrant activation of WNT/β-catenin signaling in tumor pathogenesis [5,7]. *DACT1*, a homologue of Dapper, located at chromosomal region 14q23.1, was first identified as a Dishevelled (Dvl)-associated antagonist of Wnt/β-catenin and JNK signaling pathways [8,9], *DACT1 *is expressed during embryonic development in the adult brains of mice [10,11], but studies on its role in tumorigenesis are scanty. *DACT1 *has been shown to be reduced in several tumors, such as gastrointestinal stromal tumors [12] and non-small cell lung cancer (NSCLC) [13], but overexpressed in some other tumors [14,15]. Dysregulated DACT1 was associated with poor prognosis in tumor patients [13]. *DACT1 *was also reported to be a novel inhibitor of the WNT/β-catenin signaling in hepatocellular carcinoma (HCC) [16]. However, its expression and biologic functions in breast cancer pathogenesis are unknown.

We identified *DACT1 *as a methylated target in the MB231 breast cancer cell line as compared with normal tissue in our pilot cancer epigenome study. Here, we further examined the expression and promoter methylation of *DACT1 *in multiple breast cell lines and primary tumors, and evaluated its potential as a tumor biomarker for breast cancer. We further demonstrated the biologic functions of DACT1 in breast cancer cells *in vivo *and *in vitro *in the context of the Wnt/β-catenin signaling pathway.

## Materials and methods

### Cell lines, tumor samples, and normal tissues

Several breast cancer cell lines (BT549, MDA-MB-231, MDA-MB-468, MCF-7, T47D, SK-BR-3, YYC-B1, YCC-B3, and ZR-75-1) were studied. All cell lines were maintained at 37°C in RPMI 1640 supplemented with 10% fetal bovine serum (FBS; Invitrogen, Carlsbad, CA, USA), 100 U/ml of penicillin, and streptomycin.

Normal human breast tissue RNA samples were purchased commercially (Stratagene, La Jolla, CA, USA; Millipore Chemicon, Billerica, MA, USA; BioChain Institute, Hayward, CA, USA). Primary breast tumor samples, paired surgical-margin tissues, and normal breast tissues were obtained from the First Affiliated Hospital of Chongqing Medical University, or elsewhere, as described previously [17-19]. All samples were evaluated and subject to histologic diagnosis by pathologists. Clinical information, including age, tumor grade, tumor size, follow-up data after initial diagnosis, and treatment, was obtained for the majority of tumor cases. Grading of tumors was achieved by staining with hematoxylin and eosin (H&E). All patients provided written consent for the study. The study was approved by the Ethics Committee of the First Affiliated Hospital of Chongqing Medical University (Approval notice: 2010/2012(23)).

### Semiquantitative RT-PCR analysis

Total RNA was isolated from cell lines by using TRI Reagent (Molecular Research Center, Cincinnati, OH, USA). Semiquantitative RT-PCR was performed as described previously [20]. *GAPDH *was amplified as a control. Primer sequences are listed in Table 1. RT-PCR was performed with 32 cycles for *DACT1*, and 23 cycles for *GAPDH*, by using Go-Taq (Promega, Madison, WI, USA).

**Table 1:** List of primers used in this study

PCR	Primer	Sequence (5'-3')	Product size (bp)	PCR cycles	Annealing temperature (°C)
MSP	*DACT1*m3	CGGGATAGTAGTAGTCGGC	118	41	60
	*DACT1*m4	CGCTAAAACTACGACCGCG			
	*DACT1*u3	GTTGGGATAGTAGTAGTTGGT	123	41	58
	*DACT1*u4	AAACACTAAAACTACAACCACA			
RT-PCR	*DACT1*-F	AGGAGAAGTTCTTGGAGGAG	179	32	55
	*DACT1*-R	TGAGCTAGGCCGACTGTCTG			
	*GAPDH*-F	GGAGTCAACGGATTTGGT	206	23	55
	*GAPDH*-R	GTGATGGGATTTCCATTGAT			
Real-time PCR	*DACT1*-F	GACGAGCAGAGCAATTACACC	158	40	60
	*DACT1*-R	ACCGTTTGAATGGGCAGA			
	*β-actin*-F	CCTGTGGCATCCACGAAACT	314	40	60
	*β-actin*-R	GAAGCATTTGCGGTGGACGAT			

### 5-Aza-2'-deoxycytidine and trichostatin A treatment

Cell lines were treated with 10 m*M *5-aza-2'-deoxycytidine (Aza; Sigma-Aldrich, St Louis, MO, USA) for 3 days or further treated with 100 n*M *trichostatin A (TSA; Cayman Chemical Co., Ann Arbor, MI, USA) for 14 hours.

### DNA bisulfite treatment and methylation-specific PCR

Genomic DNA was extracted from tumors, normal tissues, and cell pellets by using QIAamp DNA Mini Kit (Qiagen, Hilden, Germany). Bisulfite modification of DNA and methylation-specific PCR (MSP) were performed as described previously [21,22]. Bisulfite-treated DNA was amplified by MSP with *DACT1 *methylation-specific primer set for *DACT1 *promoter (Table 1), by using AmpliTaq-Gold DNA Polymerase (Applied Biosystems, Foster City, CA, USA). Methylated and unmethylated MSP primer sets target the same CpG sites and do not amplify genomic DNA with no bisulfite treatment.

### Quantitative reverse transcription polymerase chain reaction

Real-time PCR (rtPCR) was performed by using Maxima SYBR Green/ROX qPCR Master Mix (MBI Fermentas, St. Leon-Rot, Germany) (Table 1). Thermal-cycling reaction was performed in the 7500 Real-Time PCR System (Applied Biosystems). Melting-curve analysis and agarose gel electrophoresis of PCR products were further performed. Relative expression levels of *DACT1 *in breast tissues were standardized to β-actin levels.

### Tissue microarray and immunohistochemistry

To evaluate DACT1 expression in breast tissues, tissue microarray (TMA) was used as described previously, containing 30 pairs primary tumors and corresponding tumor-margin tissues [18]. Immunohistochemistry was performed by using a two-step method. In brief, after deparaffinization, sections were hydrated and underwent sodium citrate antigen retrieval. Sections were then incubated with 3% hydrogen peroxide to block endogenous peroxidase activity. A rabbit polyclonal antibody against human DACT1 protein (Ab104.4; Abcam, Cambridge, UK) was used. Sections were incubated with primary antibody (1:200 dilution) overnight at 4°C, detected by using polyperoxidase-anti-rabbit IgG (Jackson Immunoresearch Laboratories, West Grove, PA, USA), and counterstained with hematoxylin. To eliminate nonspecific staining, a negative control was performed with PBS.

All immunohistochemical photographs were analyzed by using Image Pro Plus (IPP, version 6.0; Media Cybernetics, Silver Spring, MD, USA), as described previously [18]. The mean optical density (OD), as a quantitative measure of stain intensity, was analyzed to determine average protein expression.

### Immunofluorescence staining

Cells transfected with pcDNA3.1-DACT1 or pcDNA3.1 plasmid were grown on glass coverslips. Transfected cells were washed with PBS, fixed with 4% paraformaldehyde in PBS for 15 minutes, permeabilized with 0.5% Triton X-100 for 30 minutes, and blocked with 3% bovine serum albumin in PBS at 37°C for 60 minutes. Cells were incubated with primary antibodies diluted in TBST at 4°C for 12 hours, washed twice with PBS, and then incubated with DyLight-conjugated anti-rabbit or anti-mouse antibody (CoWin Biotech Co., Ltd. (CWBIO), Beijing, China) for an additional 50 minutes. Nuclei were counterstained with 4,6-diamidino-2-phenylindole (DAPI) (Roche, Palo Alto, CA, USA).

### Cell-proliferation assay

MB231 cells were cultured in six-well plates at a density of 1 × 10^4 ^cells/well and allowed to grow overnight. Cultures were then transfected with pcDNA3.1-DACT1 or pcDNA3.1 plasmid by using Lipofectamine-2000 (Invitrogen). At 24, 48, and 72 hours, cell proliferation was measured by using Cell Counting Kit-8 (CCK-8; Dojindo Molecular Technologies, Inc., Kumamoto, Japan) [23].

### Colony-formation assay

Colony-formation assay was performed by using a monolayer culture. Cells (MB231 and MCF7) were plated in six-well plates and transfected with pcDNA3.1-DACT1 or pcDNA3.1 (4 μg each) plasmid by using Lipofectamine 2000 (Invitrogen). At 48 hours after transfection, cells were collected, replated, and selected for 2 weeks in the presence of G418 (0.4 mg/ml). Surviving colonies (≥50 cells/colony) were counted after staining with gentian violet. All experiments were performed 3 times.

### Wound-healing assay

*DACT1 *and *vector*-expressing cells (MB231 and MCF7) were selected by using G418, and then cultured in six-well plates until confluent. After scratching the monolayer, cells were photographed at 0, 12, 24, 36, 42, and 48 hours under a 10× objective (Leica DMI4000B, Milton Keynes, Bucks, UK).

### Caspase-3 colorimetric assay

Cells were seeded in six-well plates and transfected with pcDNA3.1-DACT1 or pcDNA3.1 plasmid, and then collected and lysed for protein purification at 24 or 48 hours. Total protein extraction was performed for analyzing active caspase-3 by using Caspase-3 Colorimetric Assay Kits (KeyGen Biotech Co, Nanjing, China).

### Western blot

Transfected cells were lysed in M-PER Mammalian Protein Extraction Reagent (Pierce, Thermo Scientific, Cramlington, UK) containing a protease inhibitor cocktail (Sigma Aldrich). A total of 50 μg of protein lysate for each sample was separated by using sodium dodecylsulfate/polyacrylamide gel electrophoresis (SDS-PAGE). The lysates were then transferred to PVDF membranes for antibody incubation. After blocking with 5% nonfat milk and 0.1% Tween 20 in TBS, the membranes were incubated with DACT1 antibody (Abcam, Cambridge, UK), or antibodies to active β-catenin (Millipore, Billerica, MA, USA), cyclin D1, c-Myc, cleaved caspase 3, and cleaved PARP (Epitomics Inc., Burlingame, CA, USA). The immunoblots were visualized by using an enhanced chemiluminescence detection system. GAPDH was used as a control.

### *In vivo *tumor model

Stable *DACT1*-expressing MB231 cells or controls (1 × 10^6 ^cells in 0.2 ml PBS) were subcutaneously injected into the right dorsal flank of female nude mice (6 weeks old, six mice per group). The weight of nude mice was measured every 7 days for 4 weeks. The xenograft tumor weight was assessed at the terminal time. The protocols for *in vivo *animal experiment were approved by the Committee on Ethical Use of Animals of the First Affiliated Hospital of Chongqing Medical University.

### Statistical analysis

Statistical analyses were performed with SPSS version 16 (SPSS Inc., Chicago, IL, USA). Student *t *test was used to analyze the difference of *DACT1 *expression between breast cancer tissues and surgical margin tissues. χ^2 ^test and Fisher Exact test were used to assess the correlation between *DACT1 *methylation and clinicopathologic parameters. For all the tests, *P *< 0.05 was considered statistically significant.

## Results

### *DACT1 *is frequently reduced in breast cancer

**Figure 1:**
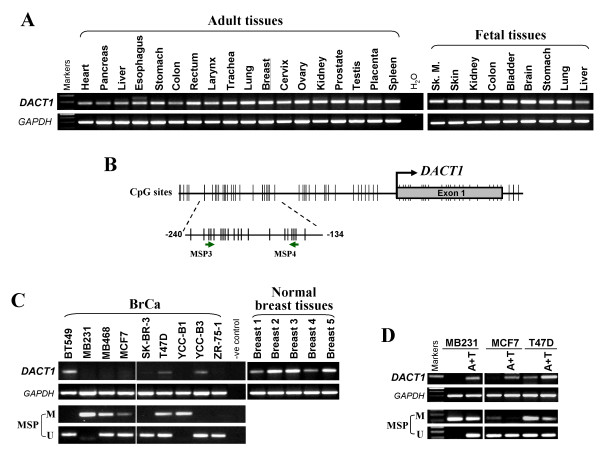
**Expression and promoter methylation of *DACT1 *in breast cancer**. **(A) ***DACT1 *expression in human normal adult and fetal tissues as detected by semiquantitative RT-PCR, with *GAPDH *as a control. **(B) **Schematic structure of the *DACT1 *promoter CpG island (CGI). Exon 1 (gray rectangle), CpG sites (short vertical lines), transcription start site (curved arrow), and MSP sites analyzed are indicated. **(C) ***DACT1 *was silenced by promoter methylation in breast cancer cell lines, but readily expressed in normal breast tissues. **(D) **Pharmacologic demethylation with Aza combined with TSA (A+T) restored *DACT1 *expression in breast cancer cell lines. M, methylated; U, unmethylated.

We first examined *DACT1 *expression in a panel of human normal adult tissues and fetal tissues, as well as breast cancer cell lines, by using semiquantitative RT-PCR. Results showed that *DACT1 *was widely expressed in human normal tissues and fetal tissues, including normal breast tissues (Figure 1A, C), but was frequently silenced or downregulated in breast cancer cell lines studied (Figure 1C).

**Figure 2:**
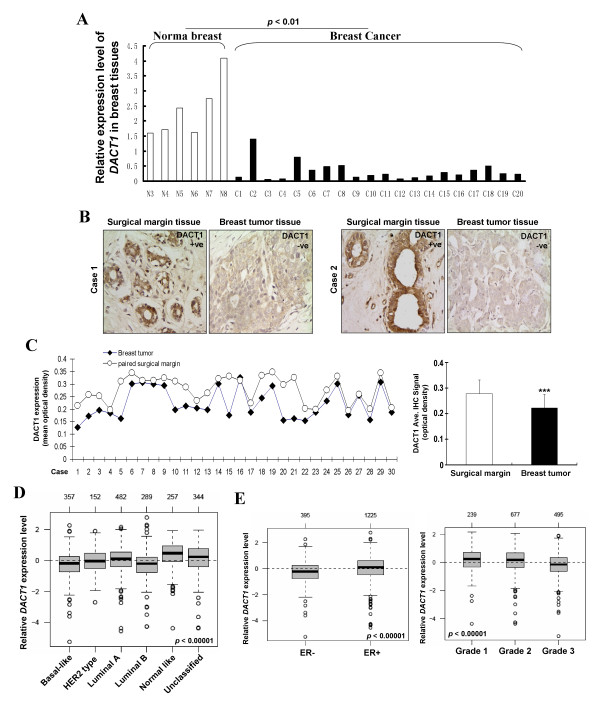
***DACT1 *was downregulated in primary breast tumors**. **(A) **Analysis of *DACT1 *expression in normal breast tissues and primary breast tumors by using real-time PCR. **(B) **Representative immunohistochemical (IHC) staining for DACT1 in paired breast tumors and surgical-margin tissues. Magnification, 400×. **(C) **The mean optical density of DACT1 expression in each case (left panel). Quantitative analysis of DACT1 expression density is shown as values of mean ± SD (right panel). ***P < 0.001. **(D, E) **GSA-tumor analysis of *DACT1 *by using the 1,881-sample breast cancer data set. Box plot of *DACT1 *expression for tumor samples stratified according to Hu subtypes, ER status, and histologic grade.

DACT1 expression in primary breast tumors was further examined at the RNA and protein levels. We found that *DACT1 *mRNA was obviously downregulated in breast cancer tissues, compared with normal breast tissues (*P < 0.01), as measured with qPCR (Figure 2A). IHC was then performed to examine DACT1 expression in 30 cases of primary tumors and paired surgical-margin tissues (Figure 2B). An IPP system showed that the mean optical density of DACT1 protein expression was significantly decreased in primary breast tumors (0.222 ± 0.060), compared with that in surgical-margin tissues (0.287 ± 0.054) (***P < 0.001) (Figure 2C).

We further assessed *DACT1 *expression by using the Gene expression-based Outcome for Breast Cancer Online (GOBO) database [24]. Gene-Set Analysis (GSA)-Tumor showed that *DACT1 *is differently expressed among different subtypes of breast cancer, with relatively high expression in a normal-like subtype (*P *< 0.00001; Figure 2D). Furthermore, decreased expression of *DACT1 *was associated with ER^- ^and higher histologic grade (*P *< 0.00001, Figure 2E). These results indicate that *DACT1 *expression is frequently downregulated in breast cancer and associated with clinicopathologic features.

### Promoter methylation of *DACT1 *contributes to its downregulation in breast cancer

We next evaluated whether *DACT1 *repression was due to promoter methylation. A typical CpG island spanning the proximal promoter and exon 1 regions of the *DACT1 *gene was found (Figure 1B). MSP showed that *DACT1 *was methylated in five breast cancer cell lines (MB231, MB468, MCF7, T47D, and YCC-B1), although no methylation was found in another two cell lines (SK-BR-3 and ZR-75-1) with silenced *DACT1 *(Figure 1C). Pharmacologic demethylation was used to assess whether promoter methylation directly regulates *DACT1 *expression. Three cell lines (MB231, MCF7, and T47D) with methylated and silenced *DACT1 *were treated with Aza and/or histone deacetylase inhibitor TSA. After treatment, *DACT1 *expression was increased, accompanied by decreased methylated alleles of *DACT1 *(Figure 1D). The results indicate that promoter methylation is a major mechanism for *DACT1 *silencing in breast cancer cells.

### *DACT1 *is methylated in breast primary tumors

**Figure 3:**
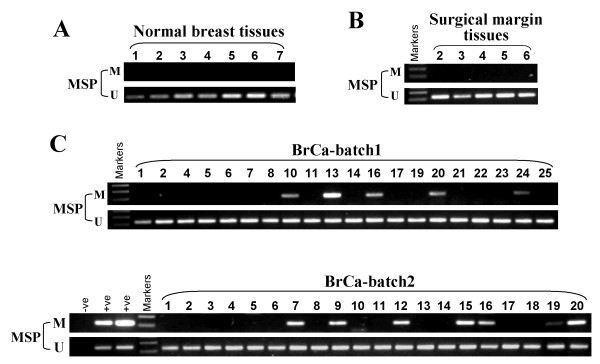
**Representative methylation-specific PCR (MSP) analysis of *DACT1 *methylation in primary breast tumors, surgical margin tissues, and normal tissues**. M, methylated; U, unmethylated.

We further investigated *DACT1 *methylation in primary tumors, surgical-margin tissues, and normal breast tissues. *DACT1 *methylation was detected in 40 (29.9%) of 134 breast cancer tissues, but not in surgical-margin tissues and normal breast tissues (Figure 3, Table 2), suggesting a tumor-specific methylation of *DACT1 *in breast cancer. We next analyzed the correlation between *DACT1 *methylation and clinicopathologic features of breast cancer patients, including age, tumor size, tumor grade, lymph node metastasis, status of estrogen receptor (ER), progestogen receptor (PR), and HER2. However, no significant correlation between *DACT1 *methylation and clinicopathologic features was found (Table 3).

**Table 2:** Promoter methylation status of *DACT1 *in primary breast tumors

Tissue	Samples (number)	*DACT1 *promoter		Frequency of methylation
			
		Methylated	Unmethylated	
Breast cancer tissues	134	40	94	40/134 (29.9%)
Breast cancer surgical-margin tissues	11	0	11	0/11 (0%)
Normal breast tissues	15	0	15	0/15 (0%)

**Table 3:** *DACT1 *methylation and clinicopathologic features of breast tumors

Clinicopathologic features		Number (*N *= 134)	*DACT1 *methylation status		*P *value
				
			Methylated	Unmethylated	
Age (years)	≤40	11	2	9	0.229
	> 40	86	30	56	
	Unknown	37	8	29	
Tumor grade	I	7	2	5	0.425
	II	56	21	35	
	III	5	1	4	
	Unknown	66	16	50	
Tumor size	≤2.0 cm	44	14	30	0.21
	≥2.0 cm, ≤5.0 cm	48	18	30	
	> 5.0 cm	4	0	4	
	unknown	38	8	30	
Lymph node metastasis	Positive	43	16	27	0.38
	Negative	41	12	29	
	Unknown	50	12	38	
Distant metastasis	Positive	1	1	0	0.114
	Negative	98	32	66	
	Unknown	35	7	28	
ER status	Positive	45	17	28	0.274
	Negative	29	9	20	
	Unknown	60	14	46	
PR status	Positive	34	11	23	0.296
	Negative	40	15	25	
	Unknown	60	14	46	
HER2 status	Positive	42	15	27	0.148
	Negative	28	11	17	
	Unknown	64	14	50	

ER, estrogen receptor; HER2, human epidermal growth factor receptor-2; PR, progesterone receptor.

### Ectopic expression of *DACT1 *inhibits breast cancer cell growth

**Figure 4:**
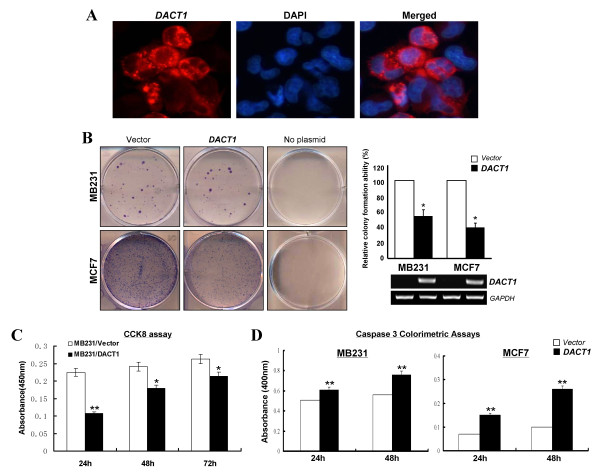
**Tumor-suppressive functions of DACT1 in breast cancer cells**. **(A) **Subcellular localization of DACT1 in MB231 cells was detected with immunofluorescence. **(B) **Representative colony-formation assay of DACT1 in MB231 and MCF7 (left panel). Quantitative analyses of colony numbers are shown as values of mean ± SD. *P < 0.05 (right panel, upper). *DACT1 *expression as measured with RT-PCR in *vector*- and *DACT1*-transfected MB231 and MCF7 cells (right panel, lower). **(C) **Cell Counting Kit-8 (CCK8) assay assessed the effect of DACT1 on cell proliferation in *vector*-, and *DACT1*- expressing MB231 cells. The values are shown as the mean ± SD. *P < 0.05; **P < 0.01. **(D) **The colorimetric assay for active caspase-3 was performed in *vector*-, and *DACT1*-expressing MB231 and MCF7 breast cancer cells at 24 and 48 hours.

*DACT1 *repression by promoter methylation in breast cancer indicated that DACT1 is likely a tumor suppressor. Immunostaining showed that DACT1 is located mainly in the cytoplasm and membrane of *DACT1*-expressing MB231 cells (Figure 4A). Colony-formation assay and CCK-8 cell-proliferation assay were further performed to assess the effect of DACT1 on cell proliferation of breast cancer. About 40% to 60% reduction of colony numbers was observed in *DACT1*-transfected MB231 and MCF7 cells, compared with controls (*P < 0.05) (Figure 4B). Cell viability was significantly decreased at 24, 48, and 72 hours after transfection with *DACT1 *in MB231 cells (**P < 0.01; *P < 0.05) (Figure 4C).

**Figure 5:**
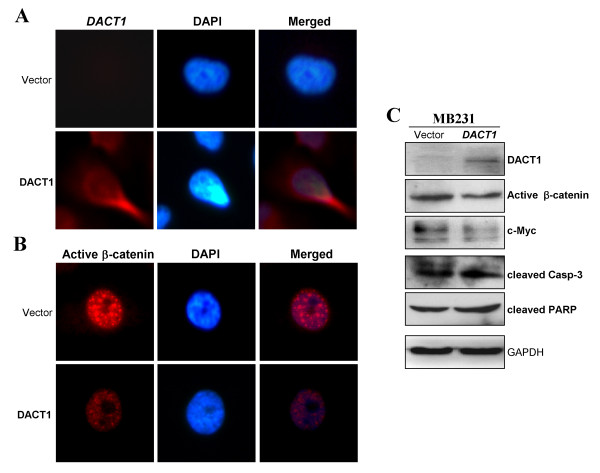
**DACT1 suppressed β-catenin activity and upregulated apoptotic markers**. **(A, B) **Subcellular location of active β-catenin in *vector*- and *DACT1*-expressing MB231 cells by immunostaining. **(C) **Western blot analysis of β-catenin signaling components and apoptotic markers.

To evaluate the molecular mechanism of DACT1 in the inhibition of cell proliferation, caspase-3 colorimetric assay was used. Results indicated that the concentration of active caspase-3 was increased in *DACT1*-expressing MB231 and MCF7 cells, compared with controls (**P < 0.01) (Figure 4D), as further confirmed by upregulated cleaved caspase-3 and cleaved PARP (Figure 5C). Thus, DACT1 is a functional TSG, inhibiting tumor cell growth and inducing cell apoptosis of breast cancer.

### DACT1 decreases β-catenin activity and suppresses breast cancer cell migration

We next investigated whether DACT1 could counteract Wnt/β-catenin signaling for its tumor-suppressive function. Expression and localization of active β-catenin were examined with immunostaining and Western blot. Reduced expression of active β-catenin and its downstream target gene *c-MYC *were detected in *DACT1*-expressing MB231 cells (Figure 5), suggesting that DACT1 antagonizes Wnt/β-catenin signaling by decreasing active β-catenin levels in breast cancer.

**Figure 6:**
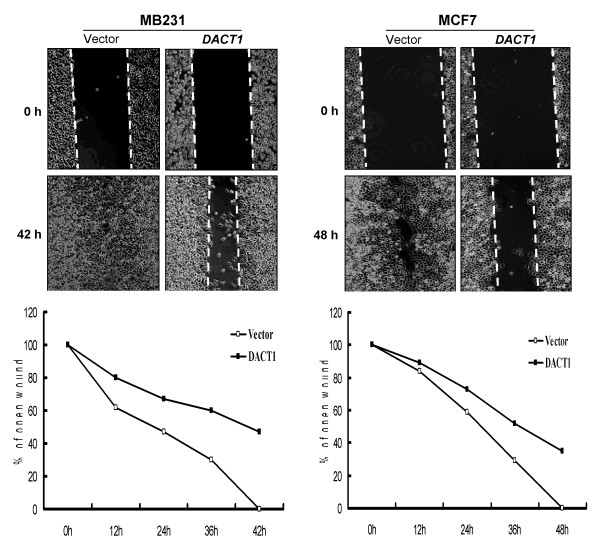
**Wound-healing assay for cell motility of *vector*- or *DACT1*-transfected MB231 and MCF7 cells**. **Upper: **Representative images of wound sealing at 0 hours and 42 or 48 hours after wound scratch. **Lower: **The percentage of wound sealing compared with that of controls at each time point as indicated.

As the Wnt/β-catenin signaling pathway plays a key role in tumor metastasis, the effect of DACT1 on cell migration was further analyzed. Wound-healing assay showed that MB231 and MCF7 cells migrated into scraped areas within 42 and 48 hours, whereas DACT1 expression decreased their wound closure by about 55% after 42 hours and about 70% after 48 hours in these two cell lines (Figure 6), suggesting that DACT1 attenuates the wound-induced cell migration of breast cancer.

### DACT1 inhibits breast tumor growth *in vivo *

**Figure 7:**
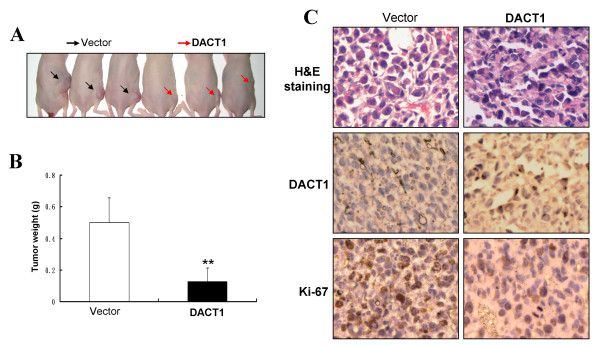
**DACT1 inhibited the tumorigenicity of breast cancer *in vivo***. **(A) **Tumors derived from *vector*- (black arrows), and *DACT1*-expressing (red arrows) MB231 cells in nude mice. **(B) **Tumor growth curve of *DACT1*-expressing cells in nude mice compared with control tumors. **P < 0.01. **(C) **Representative photographs of hematoxylin and eosin (H&E) staining and immunohistochemistry (IHC) analysis of DACT1 and Ki-67 antigen in tumors of nude mice. Magnification, 400×.

Further to evaluate the tumor-suppressive functions of DACT1 *in vivo*, tumorigenicity of MB231 cells expressing DACT1 was evaluated in nude mice. Thirty days after injection, tumors were excised from tested mice for further analysis. The average volume of tumors induced by *DACT1*-expressing MB231 cells was significantly decreased, compared with control tumors (**P < 0.01; Figure 7A, B). Immunohistochemistry was further performed to analyze the expression of DACT1 and cell-proliferation marker Ki-67 in xenograft tumors. Numerous tumor cells with higher nuclear fragmentation were observed in H&E-stained sections from *DACT1*-expressing MB231 cells compared with controls, along with decreased proliferating cells (Figure 7C). These results indicate that DACT1 does act as a tumor suppressor in breast tumorigenesis.

## Discussion

From this study, we report that *DACT1 *is widely expressed in normal breast tissues but frequently downregulated/silenced by promoter methylation in breast cancer. *DACT1 *is methylated in 29.9% of primary breast tumors, but not in surgical-margin tissues and normal breast tissues. DACT1 inhibits breast cancer cell proliferation by inducing apoptosis, and further suppresses tumor-cell migration through downregulating β-catenin activity, thus functioning as a tumor suppressor for breast cancer.

Epigenetic disruption of TSGs, including promoter methylation and histone modification, is a key mechanism regulating cancer gene expression [25,26]. *DACT1 *was frequently downregulated by promoter methylation in HCC, whereas another DACT family member, *DACT3*, was repressed by bivalent histone modifications in colon cancer [16,27]. We report that *DACT1 *was frequently methylated in breast cancer cell lines and primary tumors, which was correlated with its downregulation/silencing. No methylation was detected in some breast cell lines with silenced *DACT1*, suggesting that histone modifications or other mechanisms may be alternative mechanisms for *DACT1 *downregulation in some settings.

The Wnt/β-catenin signaling pathway plays an important role in tumor proliferation and migration [6]. Dact1 (Dapper), originally identified as an interacting protein for Dishevelled (Dvl), has been involved in distinct Wnt-dependent developmental processes of *Xenopus*, zebrafish, and mice [8,11,28-30]. Dact1 antagonizes Wnt signaling by binding with Dvl and promoting its degradation. Notably, this inhibitory activity is conserved from *Xenopus *to humans [8,9,31,32]. DACT1 has been identified disrupting the expression and localization of β-catenin, thus dysregulating Wnt/β-catenin signaling in NSCLC [13]. We found that DACT1, located mainly in the cytoplasm and membrane, reduced the expression of active β-catenin and its downstream target gene *c-MYC *in breast cancer cells, thus inhibiting cell proliferation of breast cancer by inducing apoptosis, as well as tumor cell migration.

DACT1 has been reported as a potential tumor marker associated with poor prognosis of NSCLC [13]. We observed tumor-specific methylation of *DACT1 *in breast cancer, indicating its potential as a tumor marker, although no obvious correlation between its methylation and clinicopathologic features was found, which must be further confirmed by a large sample-sized study. Future study of circulating methylated *DACT1 *in serum or in combination with other methylated TSGs may be performed for the detection of breast cancer [5,33,34].

## Conclusions

In summary, DACT1, as a Wnt/β-catenin signaling antagonist, is frequently downregulated/silenced in breast cancer, acting as a functional tumor suppressor in breast tumorigenesis, and may serve as a potential tumor marker for breast cancer.

## Abbreviations

Aza: 5-aza-2'-deoxycytidine; CGI: CpG island; DAPI: 4,6-diamidino-2-phenylindole; Dvl: Dishevelled; ER: estrogen receptor; H&E: hematoxylin and eosin; HCC: hepatocellular carcinoma; IHC: immunohistochemistry; IPP: Image Pro Plus; MSP: methylation-specific PCR; NSCLC: non-small cell lung cancer; OD: optical density; PR: progesterone receptor; qRT-PCR: quantitative reverse transcription polymerase chain reaction; SDS-PAGE: sodium dodecylsulfate/polyacrylamide gel electrophoresis; TMA: tissue microarray; TSA: trichostatin A; TSG: tumor-suppressor gene.

## Competing interests

The authors declare that they have no competing interests.

## Authors' contributions

XY, TX, WXS, XS, XL, JH, YY, and WP acquired data. XY, TX, and LL analyzed data and drafted the manuscript. MO and KK provided material. GR reviewed the manuscript. QT conceived of and supervised the study, analyzed data, and finalized the manuscript. All authors read and approved the manuscript for publication.
